# The Development of Primary Effusion Lymphoma-Like Lymphoma in a Patient with Preexisting Essential Thrombocythemia

**DOI:** 10.1155/2021/5237986

**Published:** 2021-09-11

**Authors:** Yoshiki Uemura, Marina Komoto, Mitsuo Okada

**Affiliations:** ^1^Department of Hematology, Chikamori Hospital, 1-1-16, Okawasuzi, Kochi-shi, Kochi- ken 780-8522, Japan; ^2^Department of Cardiology, Chikamori Hospital, 1-1-16, Okawasuzi, Kochi-shi, Kochi- ken 780-8522, Japan; ^3^Department of Gastroenterology, Chikamori Hospital, 1-1-16, Okawasuzi, Kochi-shi, Kochi-ken 780-8522, Japan

## Abstract

A 71-year-old Japanese male was diagnosed with essential thrombocythemia (ET) with the JAK2 V617F mutation variation, in April 2011. He was mainly treated with hydroxyurea following which the number of platelets was maintained within the normal limit. At age 80, he was hospitalized due to cardiac tamponade. Computed tomography showed no evidence of tumor masses or lymphadenopathy. Pericardial drainage was performed, and cytopathologic examination of the fluid revealed atypical lymphoid cells consistent with an effusion lymphoma of B cell lineage. The pericardial effusion was completely drained, and complete remission was achieved. Ultimately, the patient was diagnosed with primary effusion lymphoma-like lymphoma (PEL-LL). To the best of our knowledge, this is the first report of PEL-LL following ET.

## 1. Introduction

The incidence of solid cancer is 1.5–3.0 times higher in patients with myeloproliferative tumor (MPN) than in the general population. Particularly, the onset of lymphoid hematological cancer, such as non-Hodgkin lymphoma (NHL), Hodgkin lymphoma, chronic lymphocytic leukemia, and multiple myeloma, is 2.5–3.5 times higher [1].

Primary effusion lymphoma (PEL) is a rare type of high-grade B cell lymphoma localized only in body cavities, without forming masses [2]. It is caused by human herpes virus Type 8 (HHV-8) in HIV-infected patients [3]. The median overall survival for the disease course is 6–9 months [4]. On the other hand, most elderly patients with PEL are HHV-8 negative and are often administered chemotherapy. They are often cured only by body cavity drainage and generally have a good prognosis. Patients with these characteristics are distinguished from those with PEL-like lymphoma (PEL-LL) [5, 6].

We encountered a case of PEL-LL that developed during the course of treatment for the essential thrombocythemia (ET). A case of PEL-LL secondary to MPN has not been reported yet. We report this case along with a literature review.

## 2. Case Presentation

**Figure 1:**
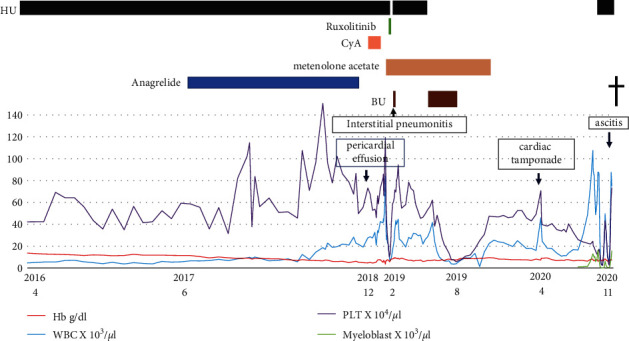
Clinical course. HU, hydroxyurea; CyA, cyclosporine; BU, busulfan; Hb, hemoglobin; WBC, white blood cell; PLT, platelets.

A 71-year-old Japanese male was diagnosed with ET, with a JAK2 V617F mutation variation, at another hospital in April 2011. The number of platelets (PLT) was maintained at approximately 50 × 10^4^/*µ*l following 500 mg–1000 mg of hydroxyurea (HU). He was transferred to our hospital in March 2016 and was continued on the same treatment. The progression of anemia and increased number of PLT were detected afterwards. We added 0.5 mg–1.0 mg of anagrelide to the 500 mg–1000 mg of HU from June 2017 and planned to stabilize the hemoglobin (Hb) level and the number of PLT. However, we could not suppress the progression of anemia by regulating the dosage of each drug. The bone marrow histology showed bone marrow hyperplasia with an increase in megakaryocytes and a significant decrease in erythroblasts in November 2018. The bone marrow chromosome analysis showed normal variation of inv(9) (p12q13) in all analyzed cells. We considered that he might have concomitant, acquired pure red cell aplasia (PRCA) without human parvovirus B19 infection and thymoma. He received 200 mg of cyclosporine (CyA) for PRCA in place of anagrelide from December 2018. The chest X-ray taken in December 2018 showed mild cardiomegaly in comparison to that of May 2017. Echocardiography revealed a large pericardial effusion. We did not perform the pericardiocentesis because he had no symptoms, such as dyspnea. He was followed up on furosemide alone. Echocardiography showed a slight decrease in pericardial effusion in January 2019. (Figure 1).

**Figure 2:**
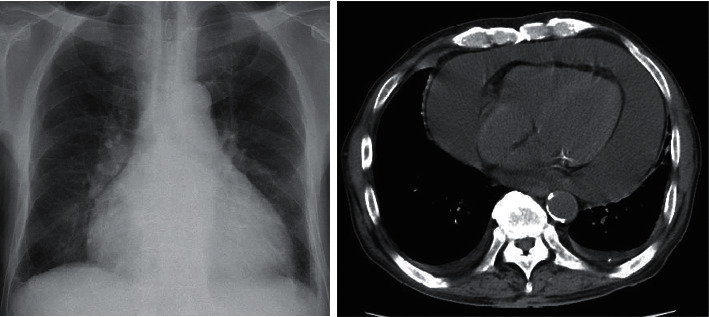
Imaging revealed heart enlargement, massive pericardial fluid, and a small amount of pleural fluid retention.

**Figure 3:**
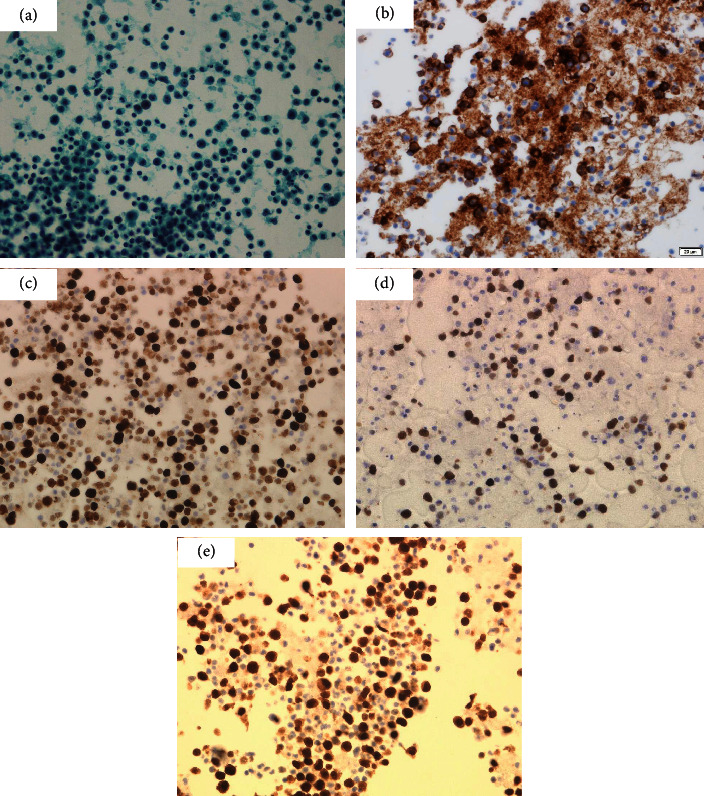
Cytological pericardial effusion findings. (a) Pap staining (×40). Cell block with ((b)–(e)) immunocytochemical findings. (b) CD20. (c) Ki-67 (MIB-1 index 50%). (d) EBER. (e) MUM-1 ((b)–(e) ×40).

**Figure 4:**
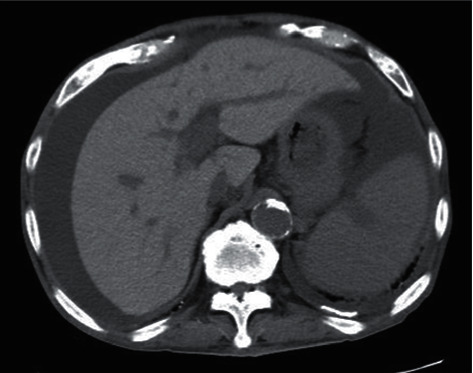
Image of plain computed tomography. Massive ascites and increased liver density.

The change from anagrelide to CyA resulted in a slight improvement in anemia. However, the PLT and white blood cell count (WBC) increased, and myeloblasts began to appear in the peripheral blood (PB). Then, we considered that he might have secondary myelofibrosis (MF), although we were unable to obtain a histopathological diagnosis because of insufficient material in the bone marrow biopsy. He was started on 10 mg ruxolitinib in place of CyA in January 2019. However, he discontinued ruxolitinib because it induced interstitial pneumonitis on the ninth day. Interstitial pneumonitis was relieved by immediate steroid therapy. He was then commenced on 10 mg metenolone acetate from February 2019 because the WBC and PLT gradually decreased. After that, WBC, PLT, and Hb levels increased rapidly. He received 2.5 mg of busulfan (BU) for cell reduction from mid-February 2019. However, he also discontinued BU because of interstitial pneumonitis on the seventh day. He then commenced HU because the WBC and PLT levels increased again after BU was discontinued. HU decreased the WBC count and the PLT; however, the anemia worsened. Therefore, the frequency of blood transfusions was increased. Afterwards, he recommenced BU from May 2019; then, the WBC count and PLT were normalized, the Hb level increased to around 8 g/dl, and then, the blast cells in peripheral blood disappeared. This time, while taking BU, there was no interstitial pneumonitis. He stopped taking BU in August 2019 due to a decrease in the WBC count and PLT. He cancelled metenolone acetate in November, 2019, because of the increase of the WBC and the PLT. After that, the WBC, the Hb level, and the PLT were kept without remarkable change. However, the WBC and the PLT soared from April, 2020. He was admitted to our hospital in April, 2020, because of severe dyspnea. He was diagnosed with cardiac tamponade caused by a large pericardial effusion (Figure 2). The examination results at the time of hospitalization are presented in Table 1. Dyspnea improved soon after the cardiac drainage. He had a class IV pericardial fluid cytodiagnosis. Immunohistochemical staining of the cell block was positive for CD20, EBER, and MUM-1, and 50% of MIB-1 (Figure 3); HHV-8 DNA was negative, and we diagnosed the patient with PEL-LL. After the drainage, the WBC count, Hb level, and PLT decreased slowly. He had a steady progression of the leg edema and showed a precipitous increase in the PB WBC and blast cell counts, from August 2020. The PLT decreased precipitously in October 2020. The bone marrow biopsy in October 2020 showed bone marrow hyperplasia with an increase in megakaryocytes and blast cells, followed by myelofibrosis of grade two or three. The blast cells occupied 32% of the bone marrow. Peroxidase staining was positive in more than 3% of the samples. Chromosomal analysis of PB demonstrated inv(3) (q21,3q26.2) in addition to inv(9) (p12q13). He was then diagnosed with acute myeloid leukemia (AML). Computed tomography (CT) showed no body cavity retention on October 1, 2020. However, massive ascites was observed on October 31, 2020 (Figure 4). Although the possibility of PEL-LL in the abdominal cavity was suggested, cytologic examination of ascitic fluid showed no malignancy. The levels of M2BPGi, type IV collagen, hyaluronic acid, and ammonia were high, and the levels of coagulation factors, albumin, and cholinesterase, from the liver, were low. Furthermore, CT and ultrasonography showed no features of cirrhosis. The CT value of the liver increased because of iron overload caused by the frequent blood transfusion. Although 5.1 L of ascites was drained at once, it reaccumulated immediately. Even after cell-free and concentrated ascites reinfusion therapy (CART), the ascites reaccumulated immediately. Subsequently, he worsened renal dysfunction and hyperammonemia and finally died of pneumonia in November 2020.

## 3. Discussion

Patients with MPN such as polycythemia vera (PV) or ET are at a high risk of solid carcinoma. Brabrand and Frederiksen demonstrated that the incidence of a new malignant tumor is 1.5–3.0 times higher in patients with MPNs [1]. The frequency of malignant lymphoma, skin cancer, lung cancer, renal cancer, and thyroid cancer is relatively high in new malignant tumors. The frequency of lymphoid hematological cancer is 2.5–3.5 times higher than those of other cancers [1]. The cause of the high frequency of secondary cancers in MPN is still unclear. The participation of shared risk factors, inherent tendency to cancer, and antitumor treatment are suspect [7–14]. Antitumor agents for treating MPN influence the risk of transformation to AML [10, 15]; however, it is unknown whether these agents influence the risk of solid carcinoma and lymphoid tumor. Two cohort studies recently reported that the incidence of aggressive B cell lymphoma in the MPN patients who received JAK1/2 inhibitor is approximately 15 times that of healthy persons [16]. Because the occurrence of pericardial effusion was detected before ruxolitinib and the dosing period was short (nine days), we excluded ruxolitinib as a cause of PEL-LL. However, ruxolitinib may promote the development of pericardial fluid. Generally, most patients treated with JAK inhibitors have previously received HU or alkylating agents. This suggests that the cumulative phenomenon of immunosuppression or insufficiency of immune surveillance could also be a cause of lymphoproliferative neoplasm (LPN). Our patient had also received HU long-term and BU and CyA short-term, prior to the administration of ruxolitinib. These preceding agents could exacerbate the risk of LPN. MPN is recognized as a model of inflammatory disease in which cytokines play an important role in the onset and progression of the disease [17]. The mechanism of NHL by JAK2 inhibitors is largely still unknown in MPN. JAK2 inhibitors damage the natural killer cells and dendritic cells quantitatively and functionally and decreases the regulatory T cells (Tregs), resulting in the suppression of inflammatory cytokine secretion mediated by CD4-positive T lymphocyte [18–20]. Thus, ruxolitinib is antagonistic towards immunological surveillance. Generally, the PLT in ET can be controlled well by HU.

The management of PLT by HU was difficult because of the progression of anemia in our case. As previously shown, erythroblasts decrease while granulocytic cells and megakaryocytes increase at the early stage of myelofibrosis [21]. Bone marrow fibrosis was not observed when the bone marrow fluid was aspirated. Although he was commenced on CyA in consideration of PRCA, the anemia did not improve. We reported that PRCA developed as ET progressed [22]. As previously considered, the onset of PRCA in patients with MPN is caused by a diverse proliferation of MPN stem cells and a diverse reaction of MPN stem cells to growth factors; however, this is not based on an immunological foundation. Therefore, the previous study finding did not support the efficacy of CyA for PRCA in patients with MPN.

We first speculated that the acute development of ascites might indicate the possibility of PEL-LL. However, cytodiagnosis of ascites showed no malignancy. Papanicolaou staining did not reveal any lymphoma cell. May–Grünwald–Giemsa (May–Giemsa) staining could not prove any lymphoma cells. Flow cytometry did not reveal the monoclonality of B cell. In addition, the ascites reaccumulated promptly after drainage. Therefore, we thought that the ascites were not derived from PEL-LL. Since CT showed a smooth liver surface, although the CT value was high due to transfusional iron overload, we rejected liver cirrhosis as the cause of the ascites. We hypothesized that decreased protein synthesis due to liver fibrosis or hepatic infiltration with leukemia cells might cause ascites.

## 4. Conclusion

We encountered a case of PEL-LL with severe cardiac tamponade throughout the progression of ET. If an unidentified pericardial effusion is detected during the course of MPN, it is important to consider PEL-LL as a differential diagnosis.

## Figures and Tables

**Table 1:** Laboratory findings.

Complete blood		Count biochemistry		Test serological test	
WBC	46,100/*μ*l	TP	7.1 g/dl	C3	66 mg/dl
Myeloblast	(+)H	Alb	3.8 g/dl	C4	19 mg/dl
Myelocyte	2.0%	AST	15 U/l	ANA	<40
Metamyelocyte	0.5%	ALT	23 U/l	RF	<5 IU/ml
Banded neutrophil	7.0%	ALP	281 IU/ml	BNP	125 pg/ml
Segmented neutrophil	78.5%	*γ*-GTP	31 IU/l	CK-MB	mg/dl
Lymphocyte	3.0%	LDH	208 U/l	MB/CK	52%
Monocyte	6.5%	T-bil	1.0 mg/dl	Troponin T	0.012
Eosinophil	1.5%	BUN	65.1 mg/dl	HCV ab	(−)
Basophilic	1.0%	Cr	2.36 mg/dl	HBs Ag	(−)
RBC	227 × 104/*μ*l	UA	16.9 mg/dl	IgG	1316 mg/dl
Hb	7.3 g/dl	T-CHO	104 mg/dl	IgA	255 mg/dl
Ht	22.5%	TG	60 mg/dl	IgM	114 mg/dl
MCV	99.1 fl	Glu	220 mg/dl	sIL-2R	1920 U/ml
Plt	70.8 × 104/*μ*l	HbA1c	6.4%		
		Na	128 mEq/l	*Coagulation*	
*Urinalysis*		K	4.8 mEq/l	PT-INR	1.52
Color	Yellow	Cl	95 mEq/l	APTT	57.9 sec
Protein	1(+)	CRP	3.73 mg/dl	Fibrinogen	272.3 mg/dl
Glucose	(−)	ESR	2 mm/h	FDP	3.0 *μ*g/ml
Occult blood	1(+)			D-dimer	1.5 *μ*g/ml
				*Quantitation of viral DNA*	
				HHV-8 DNA < 100 copy/ml	

## Data Availability

All data are available in the hospital's medical records.
